# Association of rs1137101 polymorphism in LEPR and susceptibility to knee osteoarthritis in a Northwest Chinese Han population

**DOI:** 10.1186/s12891-016-1162-0

**Published:** 2016-07-25

**Authors:** Jianhui Yang, Heng Du, Jianguo Lv, Lianhe Zhang

**Affiliations:** 1 Rehabilitation Center, First Affiliated Hospital of Xi’an Jiaotong University Health Science Center, No. 277, Yanta West Road, Xi’an, 710061 Shaanxi Province People’s Republic of China; 2 Department of Orthopedics, First Affiliated Hospital of Xi’an Jiaotong University Health Science Center, Xi’an, 710061 Shaanxi Province People’s Republic of China; 3 Department of Statistics, Xi’an Jiaotong University Health Science Center, Xi’an, 710061 Shaanxi Province People’s Republic of China

**Keywords:** LEPR, SNPs, Knee OA, Gln223Arg

## Abstract

**Background:**

Osteoarthritis (OA) is a complex arthritic condition in which the genetic factor plays a crucial role. A single nucleotide polymorphism (SNP), rs1137101 (Gln223Arg) of leptin receptor (LEPR) gene has been demonstrated to be associated with susceptibility to knee OA. However, this association in Chinese Han population has never been examined. The present study aimed to investigate whether Gln223Arg was related to knee OA susceptibility in a Northwest Chinese population with Han ethnicity.

**Methods:**

Gln223Arg polymorphisms were genotyped in 587 patients with confirmed knee OA and in 628 age- and sex-matched healthy controls using polymerase chain reaction-restriction fragment length polymorphism analysis. Besides, *LEPR* genotypes were verified by direct DNA sequencing analysis on PCR products.

**Results:**

The genotype and allele frequencies in *LEPR* SNP rs1137101 were significantly different between cases and control groups (chi-square = 6.52, *P* = 0.038 for genotype and chi-square = 5.06, *P* = 0.024 for allele frequencies; respectively). Rs1137101 was correlated with knee OA in the dominant genetic model (GG + GA versus AA) (*P* = 0.016) and a higher G allele frequency existed (*P* = 0.024) among all patients with knee OA and controls. On stratification analysis, the genotype GG and G allele were associated with susceptibility to knee OA in females, both young (≤65 years) and old groups (>65 years) and patients with mild knee OA.

**Conclusions:**

Our finding suggested that the genetic variant of *LEPR* gene rs1137101 is independently related to knee OA susceptibility in Northwest Chinese population with Han ethnicity and may serve as a potential biomarker to determine risk of knee OA.

## Background

Osteoarthritis (OA) is a common form of progressive joint disorder characterized by age-related regressive changes in articular cartilage, with joint space narrowing, osteophyte formation and subcondral sclerosis [1, 2]. Common symptoms of OA consists of severe joint pain, stiffness, swelling, reduced motion, and a reduced quality of life. Among different joints part in the human body, the knee is the most common prevalent site that primary OA might be involved. A recent survey reported the morbidity of primary knee OA in people over 60 years is 19.4 % in China [3]. Although the exact causes of this disease were still not well-defined till now, it has been demonstrated to be associated with several attributing factors including a variety of non-genetic factors (obesity, sex, age, and joint injury) and a combinatorial effect of genetic factors [4–6]. Therefore, there is an attractive prospect for us to find a susceptibility gene of knee OA, which could not only bring us a better understanding of the molecular mechanism of the disease, but also benefit for the development of OA targets drugs.

Leptin, a endogenous hormone expressed by the *obese* gene mainly in adipocytes, functions to control the metabolism of glucose and lipid through binding to the leptin receptor (LEPR, also known as Ob-R) [7]. Dumond et al. [8] firstly reported the expression of leptin in articular cartilage and synovial fluid (SF), with only few leptin expression in normal cartilage chondrocytes, while in osteoarthritic cartilage and the areas of osteophytes formation, the expression level of leptin was remarkably increased and closely related to the severity of cartilage damage. Simopoulou et al. [9] demonstrated that leptin could induce the expression of metalloproteinases 9 and 13, 2 major enzymes that mediate the destructive process in OA and production of inflammatory cytokine IL-1β in chondrocytes, which contributes to the development and progression of OA. Ku et al. [10] reported that SF leptin concentrations in patients with knee OA was positively associated with Kellgren/Lawrence (K/L) grading and could be used as an effective marker for quantitative detection of OA severity. All these results intensively suggested that leptin mediated a detrimental effect on cartilage metabolism and may thus play potential roles in the pathogenesis of OA.

LEPR is a membrane-spanning glycoprotein belonging to the class I cytokine-receptor family which mediates the effects of over 20 kinds of cytokines. Among the widely expressed alternative spliced isforms (including Ob-Ra, Ob-Rb, Ob-Rc, Ob-Rd, and Ob-Re), the long isoform Ob-Rb is the only 1 indentified to be functional [11]. Recent study showed that LEPR was also expressed within the native human cartilage chondrocytes, mediating the inflammatory and destructive responses of leptin in cartilage and other joint tissues [12]. Simopoulou et al. [9] detected the expression of LEPR in articular cartilage of controls and patients with minimal OA and demonstrated the expressions of LEPR mRNA and protein were both significantly increased in advanced osteoarthritic cartilage compared to that of patients with minimal OA and normal controls. Since LEPR is of functional importance for the risk factor of OA-obesity, numerous investigations regarding the association between *LEPR* SNPs with obesity have been extensively carried out [13–16]. Among the various SNPs of *LEPR*, Gln223Arg (rs1137101) variant of LEPR exon 4 received more attention. By conducting a population-based case–control study, Portoles et al. [13] discovered the Gln223Arg polymorphism was significantly associated with obesity in Spain population. Other studies demonstrated that the Gln223Arg polymorphism is highly related to obesity in Pacific Islanders [14], body mass index (BMI), body fat amount, serum leptin concentrations in Caucasian woman [15] and antipsychotic-induced body weight gain [16].

Although there have been studies demonstrating the association of *leptin* gene SNPs with knee OA susceptibility [17] and it seems that these *LEPR* polymorphisms are associated with risk factors for knee OA, yet few studies have been conducted directly regarding the association of *LEPR* SNPs and susceptibility to knee OA. In 2003, Ma et al. [18]. genotyped *LEPR* SNP A668G (Gln223Arg) in 148 patients with knee OA and 155 controls, concluding that Gln223Arg is associated with susceptibility to primary knee OA in Ningxia Hui population, but this association in Chinese Han population still remains unknown. Therefore, the present case − control study aims to investigate whether the genetic polymorphisms of Gln223Arg in *LEPR* gene is associated with OA susceptibility in Northwest Chinese population with Han ethnicity.

## Methods

### Study population

The procedure of the study was reviewed by the Ethical Review Committee of Health Science Center, First Affiliated Hospital of Xi’an Jiaotong University and conducted in line with the Declaration of Helsinki. Written informed consent was obtained from all participants prior to their participation in the study. A total of 1 215 subjects were recruited in this study, consisting of 587 patients (134 males and 453 females) with confirmed knee OA and 628 age − and sex − matched healthy controls (143 males and 485 females). The diagnosis of OA was based on the symptomatic criteria of American College of Rheumatology and radiographic signs (K/L grading ≥ 2) for OA [19] of at least 1 knee. The patients were recruited at the Department of Orthopaedics, First Affiliated Hospital of Health Science Center, Xi’an Jiaotong University between January 2010 and December 2014. Controls with normal knee radiographs (K/L grading < 2) undergoing physical examination were consecutively collected in a large member of individuals contemporaneously. Participants were excluded on the basis of having arthropathy due to gout, pseudogout, rheumatoid arthritis, with histories of corticosteroid medication, bilateral knee replacements, other forms of arthritis, cancer, or other chronic inflammation diseases. All individuals enrolled in the present study were drawn from a local Chinese Han ethnic population of Shaanxi Province in Northwest China. All subjects were stable residents in the areas and no 1 dropped out during the process of study.

### Genotyping

Venous blood samples were collected from all subjects with informed consent after overnight fasting. Genomic DNA was all extracted from blood samples with the commercially available DNA extraction kit (TIANGEN Biotech, Bejing, China) according to the manufacturer’s instructions and stored at − 80 °C until utilized. The genotypes of Gln223Arg polymorphism in *LEPR* gene were determined by polymerase chain reaction-restriction fragment length polymorphism (PCR-RFLP) methods. Sequencing primers for PCR were designed with Primer Express 3.0 software (Applied Biosystems, Waltham, MA). The primers used for amplification were: Forward 5′-TCCTGCTTTAAAAGCCTAATCCAGTATTT-3′ and Reverse 5′-AGCTAGCAAATATTTTTGTAAGCAAT-3′ for Gln223Arg. The PCR reaction was performed on 25 μL reaction mixture containing 4 μL mixed DNA template, 1 μL of each primer, 12.5 μL 2 × Reaction Mix and 6.25 μL ddH_2_O. The PCR protocol was carried out as followed: initial denaturation at 95 °C for 4 min followed by 30 cycles of 94 °C for 30 s, annealing at 59.5 °C for 30 s, 72 °C for 30 s, and a final extension at 72 °C for 7 min. PCR products were digested over night at 37 °C with 5U of *Msp*I restriction enzymes (Thermo Scientific, Waltham, MA, USA) for Gln223Arg polymorphisms. The digested mixtures were then separated on a 2.0 % agarose gel and visualized by ethidium bromide staining and ultraviolet light transillumination. The expected fragment of Gln223Arg was 242 bp and 125 bp in AA, 367 bp in GG and 367, 242 and 125 bp in AG genotypes, respectively. Fifty percent of the samples of both cases and control subjects were randomly selected for reproducibility tests at least twice in different days by using direct DNA sequencing with ABI3500DX Genetic Analyzer (Thermo Scientific, Waltham, MA, USA) to verify the accuracy of PCR-RFLP method.

### Statistical analysis

Statistical analyses were performed using SPSS software, version 16.0 for windows (SPSS Inc.; Chicago, IL, USA). Baseline quantitative data are represented as mean ± SD. The Chi-square goodness-of-fit test was employed to assess the Hardy-Weinberg equilibrium (HWE) for genotype and allelic proportions in the cases and control groups. The associations between the Gln223Arg polymorphism and the risk of knee OA was evaluated with odds ratios (ORs) and 95 % confidence intervals (CI) after adjusting for age and BMI with multivariate logistic regression analysis. Further stratifications analysis for sex, age and the radiographic severity of OA were subsequently conducted. Kolmogorov-Smirnov test was performed to analyze the data normality and unpaired *t* test, Mann–Whitney *U* test, or chi-square test was used to assess significance in clinical characteristics between patients with knee OA and healthy controls, as appropriate. Clinical characteristics of OA patients with different genotype were compared using chi-square or Kruskal-Wallis tests. For all the tests, *P* < 0.05 was considered as statistically significant. The power of the study was estimated with the published data in the literature and then calculated with Power and Precision software (Biostat, Englewood, NJ, USA), with results reaching over 75 %.

## Results

### Baseline clinical parameters of the study groups

A total of 1 215 Northwest Chinese subjects with Han ethnicity were recruited in this case–control study, comprising 587 patients with knee OA (134 males and 453 females; mean age 61.37 ± 10.64 years) and 628 healthy controls (143 males and 485 females; mean age 60.46 ± 9.85 years). The baseline characteristics of both groups were summarized in Table 1. No significant differences in age and gender were found between the 2 groups (*P* > 0.05). Similar with the previous studies that reported high BMI increased the risk of developing OA [20], BMI was found significantly higher in patients group compared to control group (*P* < 0.001). According to the radiographic KL grading system, approximately 50.3 % of the patients had a K/L grading of 3 or 4. There were no significant differences in the age, gender and BMI between different K/L grading subgroups (*P* > 0.05).

**Table 1:** The general characteristics of study subjects

Characteristics	Knee OA patients (*n* = 587)	Healthy controls (*n* =628)	*P*-value
Age (years)	61.37 ± 10.64	60.46 ± 9.85	0.537
Gender (F/M)	453/134	485/143	0.981
BMI (kg/m^2^)	26.31 ± 4.45	24.75 ± 4.16	<0.001
K/L grades			
Grade 2, *n* (%)	292 (49.74)	0 (0.0)	—
Grade 3, *n* (%)	175 (29.81)	0 (0.0)	—
Grade 4, *n* (%)	120 (20.45)	0 (0.0)	—

### Genotypic distribution and allelic frequencies of Gln223Arg polymorphism

The results of the genotype distribution and allele frequencies of *LEPR* Gln223Arg for cases and control groups were presented in Table 2. *LEPR* Gln223Arg variant genotype frequencies in either the cases or the control group was found to be in Hardy–Weinberg equilibrium (*P* = 0.85 and 0.83, respectively). The genotype GG and allele-G were the most common genotype and allele in both cases and control groups. The allele frequencies of Gln223Arg in cases group (G, 68.57 %; A, 31.43 %) were remarkably different from those of control group (G, 64.25 %; A, 35.75 %, chi-square = 5.06, *P* = 0.024). Besides, the genotype distributions of Gln223Arg in cases group (GG, 45.49 %; GA, 46.17 %; AA, 8.35 %) were also significantly different from those in control group (GG, 41.08 %; GA, 46.34 %; AA, 12.58 %; chi-square = 6.52, *P* = 0.038).

**Table 2:** The genotype and allele frequencies of rs1137101 polymorphism of LEPR gene in knee OA patients and healthy controls

Groups	Number	Genotype frequencies (%)			Allele frequencies (%)		χ2	*P*-value
		GG	AG/GA	AA	G	A		
Cases	587	267 (45.49)	271 (46.17)	49 (8.35)	805 (68.57)	369 (31.43)	2.96	0.85
Control	628	258 (41.08)	291 (46.34)	79 (12.58)	807 (64.25)	449 (35.75)	0.05	0.83
Total	1215	523 (43.05)	571 (47.00)	121 (9.96)	1617 (66.54)	813 (33.46)	1.55	0.21
χ2 = 6.52, *P* = 0.038 2 = 5.06, *P* = 0.024								

### Association of Gln223Arg polymorphism and susceptibility of knee OA

Table 3 illustrates the genetic association between the Gln223Arg polymorphism of *LEPR* gene and risk of knee OA. The data demonstrated that the Gln223Arg genetic polymorphism of *LEPR* gene was significantly related to the susceptibility of knee OA in patients as a whole and controls in the dominant model (GG + GA versus AA) (OR = 1.58; 95 % CI, 1.09–2.30; *P* = 0.016) and G allele was correlated with the higher risk of knee OA (OR = 1.21; 95 % CI, 1.03–1.44; *P* = 0.024). When further stratified by sex after adjusting for other variables such as age and BMI, multivariate logistic regression analysis showed the evident differences of genotype distribution in female subjects (OR = 1.89; 95 % CI, 1.25–2.87; *P* = 0.008). Additionally, G allele carriers were still related to the increased risk of knee OA in females (OR = 1.56; 95 % CI, 1.08–2.49; *P* = 0.012). However, both of the genotype distributions and allele frequencies in males were found no significant differences. When stratification by age, both young (≤65 years) (OR = 1.48; 95 % CI, 1.18–2.36; *P* = 0.028) and old (>65 years) (OR = 1.72; 95 % CI, 1.13–2.53; *P* = 0.020) patients showed significant differences in combined genotype (GG + GA) versus AA after adjustment for BMI and gender compared to control group. Besides, G allele carriers suffer higher risk of knee OA in both young (OR = 1.26; 95 % CI, 1.04–1.58; *P* = 0.045) and old patients (OR = 1.38; 95 % CI, 1.13–1.57; *P* = 0.037). After stratification according to the radiographic severity of knee OA, significant differences were still found to be existed in the comparison of combined genotype (GG + GA) versus AA after adjusting for variables such as age, BMI, and gender in cases with mild knee OA (OR = 1.53; 95 % CI, 1.05–2.47; *P* = 0.022). Additionally, G allele was moderately associated with an increased risk of knee OA in mild subgroup (OR = 1.30; 95 % CI, 1.05–1.62; *P* = 0.013). Unexpectedly, we didn’t detect any association between genotype distributions or allele frequencies of rs1137101 and the risk of knee OA in severe subgroup.

**Table 3:** Association of the rs1137101 polymorphism of LEPR gene with risk of knee OA with stratifications by age, gender and K/L grades

Groups compared	GG + GA versus AA			GG versus AG + AA			G allele versus A allele		
	OR	95 % CI	*P*-value	OR	95 % CI	*P*-value	OR	95 % CI	*P*-value
All patients (*n* = 587) versus all controls (*n* = 628)	1.58	1.09—2.30	0.016	1.20	0.95—1.50	0.12	1.21	1.03—1.44	0.024
Gender groups									
Male (124 patients vs. 143controls)	0.82	0.52—1.28	0.33	1.13	0.44—2.56	0.71	0.90	0.55—1.36	0.46
Female (453 patients vs. 485 controls)	1.89	1.25—2.87	0.008	1.28	0.85—1.75	0.53	1.56	1.08—2.49	0.012
Age groups									
≤65 years (285 patients vs. 278 controls)	1.48	1.18—2.36	0.028	0.95	0.68—1.32	0.65	1.26	1.04—1.58	0.045
>65 years (302 patients vs. 350 controls)	1.72	1.13—2.53	0.020	1.13	0.84—1.56	0.18	1.38	1.13—1.57	0.037
K/L grading groups									
Mild OA (292 patients vs. 628 controls)	1.53	1.05—2.47	0.022	1.08	0.86—1.29	0.31	1.30	1.05—1.62	0.013
Severe OA (295 patients vs. 628 controls)	1.05	0.67—1.64	0.84	0.95	0.70—1.30	0.73	0.98	0.78—1.25	0.91

*OR* odds ratio, *CI* conidence interval; vs. versus

AA, AG, and GG genotypes were grouped together and a 2 × 3 contingency-table analysis was performed

## Discussion

Identification of patients at risk for incident disease or disease progression in OA remains challenging, because the right now preferred radiography method is an insensitive reflection of genetic changes that presage cartilage and bone abnormalities. Although the etiology of OA is multifactorial, there have been compelling evidence suggesting the involvement of genetic factors in the pathogenesis of OA [21]. Thus, there is a widely appreciated need for identification of susceptibility genes of OA. In recent years, many studies have been intensively carried out to determine the related genes that may cause knee OA [6, 22–24]. Among which, the impaired leptin signal pathway was demonstrated to be highly related to the pathophysiology of OA. Considering the crucial role of LEPR in regulating leptin activity, here we investigated the effects of the common exonic polymorphism of Gln223Arg in *LEPR* gene on the risk of knee OA in Northwest Chinese population with Han ethnicity.

The present case–control study firstly examined whether the *LEPR* SNP polymorphism was correlated with the presence of primary knee OA in Northwest Chinese population. Our results indicated that *LEPR* gene SNP rs1137101 (Gln223Arg) polymorphism was correlated with risk of knee OA. We have found GG genotype distribution and G allelic frequencies of Gln223Arg were both significantly higher in OA patients than that of control group, and G allele of rs1137101 is a common susceptibility allele for knee OA at least in the Han population in Northwest China. Multivariate logistic regression indicated that rs1137101 was associated with knee OA in the dominant genetic model (GG + GA versus AA) and G allele carriers of Gln223Arg had higher risks of knee OA, which is consistent with reports from Spanish Mediterranean population and Pacific Islanders [13, 14]. These 2 studies both detected the significant association of Q223R (Gln223Arg) polymorphism and prevalence of obesity in the dominant model for Q223R (QQ + QR versus RR) and revealed a significant association between the 223Q carrier genotype and obesity [13, 14]. However, the population we examined here had a BMI in a normal range (not obese) and significant associations of Gln223Arg and risk of knee OA were still detected, which indicates this association is not the only risk factor for knee OA in the Chinese Han population and implies the possibility that the effects of Gln223Arg appear in correlating with other genetic or environmental factors. Therefore, further studies are needed to investigate the potential role of Gln223Arg for predicting risk of knee OA in Chinese Han population in the absence of obesity.

On stratification analysis, the G allele carriers (GG + GA) and G allele were related with knee OA susceptibility in females, both young (≤65 years) and old groups (>65 years) and patients with mild knee OA after adjustment for other potential confounders such as age, gender and BMI. But the association between the risk of knee OA and rs1137101 G allele carriers (GG + GA) was stronger in female patients (1.72-fold) and those >65 years old (1.89-fold) compared with AA homozygote. However, it should be noted there were no associations between Gln223Arg polymorphism and knee OA susceptibility in male participants and patients with higher K/L grading. We assume that the disappearance of association in male participants may be ascribed to the relative small sample size and/or sex disparities in the genetics of OA. Although sex disparities in the genetics of OA have not been fully understood, in addition to the effect of estrogen, a higher incidence and heritability of knee OA has been reported for women than for men [5, 25–27], as well as sex differences in cartilage volume [28]. The lack of difference in patients with higher K/L grading can be attributed to the possibility that rs1137101 might just play important roles in knee OA induction (with relative lower K/L grading), not in the radiographic progression of OA and that rs1137101 may be in linkage disequilibrium with some other more relevant alleles within the *LEPR* gene region. Previous studies of candidate genes for OA demonstrated different SNPs may function differently in the induction and progression of knee OA. *ADAM12* gene was found to be involved in both the induction and radiographic progression of knee OA [29]. *NCOR2* and *CD36* genes were associated with induction of radiographic knee OA measured as either a K/L grading of ≥2.0 or the presence of osteophytes [29], while the polymorphisms at *CILP*, *TNA*, *IL1RN* and variation at inflmmatory genes appeared to correlate with radiographic progression over time [29–31]. Besides, mitochondrial DNA haplogroups are also associated with the induction and progression of knee OA [32]. Thus, polygenic effects on the pathogenesis of knee OA should not be ignored and there exists a necessity to explore other potentially contributing genes involved in the development of knee OA for a better comprehension of genetic regulation on knee OA.

Originally, the SNP rs1137101 designates a change of glutamine to an arginine at codon 223 (Gln223Arg) in the extracellular domain coding region of the LEPR protein [33]. This transition results in an exchange of a neutral amino acid for 1 with a positive charge, altering receptor structure and function and affecting signaling capacity and circulating leptin levels [34]. Previous studies have mostly reported the association of Gln223Arg polymorphism and risk of different kinds of cancers [34–36] and cardiovascular disease [37–39]. Gln223Arg was recently recognized as a risk predictor of knee OA in Ningxia Hui population, however, there have been no detailed data concerning the association *LEPR* SNP rs1137101 with risk of OA in Chinese Han population thus far. We were the first to show that Gln223Arg polymorphism was correlated with risk of knee OA in Northwest Chinese population with Han ethnicity. The correlation between the Gln223Arg polymorphism and knee OA susceptibility could be possible interpreted by physiological role of leptin. As described previously, leptin can also be produced by many other tissues and cells other than adipose tissue, such as joint tissues [8, 10]. Besides, LEPR was also expressed within the native human cartilage, mediating the inflammatory and destructive responses of leptin in cartilage and other joint tissues [12]. Further mechanical studies indicated that the Gln223Arg polymorphism might affect leptin signaling through phosphorylation of signal transducers and activators of transcription 3 (STAT3) [40] and was in linkage disequilibrium with other functional polymorphisms that can impair the signaling capacity of the LEPR [41]. The G to A mutation in rs1137101 led to reduced proliferation and synthesis of extracellular matrix and an amelioration of the anti-apoptotic effect of leptin on articular chondrocytes and eventually causes OA happen.

However, we have been aware of several potential limitations existing in this study that could affect our conclusions. First, as this was a study comprising a relatively small number of subjects, it is difficult to draw a strong conclusion. Further studies with a larger population sample are warranted to substantiate our results. Second, the *P*-value (*P* = 0.537) for age-matching between patients and healthy controls appeared to be marginal, which might affect our ability to detect the association between Gln223Arg polymorphism and the susceptibility of OA to some extent. Third, we have just focused on the association and missed the aspects of the functional study of *LEPR* gene polymorphism, which might potentially neglect other gene SNPs that may strongly correlate with OA. Lastly, we did not analyze the relationship between Gln223Arg polymorphism and joint pain experienced by patients. Further studies are warranted to detect the role of Gln223Arg polymorphism for predicting joint pain in patients with knee OA.

## Conclusions

In conclusion, this study firstly demonstrated that the *LEPR* rs1137101 (Gln223Arg) polymorphism was associated with the susceptibility of knee OA in the dominant genetic model (GG + GA versus AA) in Northwest Chinese population with Han ethnicity. This association remained in patients with mild radiographic severity and female subjects. Further studies with larger-scale population and different ethnicities should be conducted to substantiate the current findings.

## Abbreviations

95%CI, 95 % conidence intervals; BMI, body mass index; K/L, Kellgren/Lawrence; LEPR, leptin receptor; OA, osteoarthritis; Ors, odds ratios; PCR-RFLP, polymerase chain reaction-restriction fragment length polymorphism; SNP, single nucleotide polymorphism
